# Measurement properties of the German version of the Physical Activity Enjoyment Scale for adults

**DOI:** 10.1371/journal.pone.0242069

**Published:** 2020-11-18

**Authors:** Darko Jekauc, Carina Nigg, Claudio R. Nigg, Markus Reichert, Janina Krell-Roesch, Doris Oriwol, Steffen Schmidt, Kathrin Wunsch, Alexander Woll

**Affiliations:** 1 Institute of Sports and Sports Science, Karlsruhe Institute of Technology, Karlsruhe, Baden-Wuerttemberg, Germany; 2 Department of Psychiatry and Psychotherapy, Central Institute of Mental Health, Medical Faculty, Mannheim, Heidelberg University, Mannheim, Baden-Wuerttemberg, Germany; Iranian Institute for Health Sciences Research, ISLAMIC REPUBLIC OF IRAN

## Abstract

The physical activity enjoyment scale (PACES) is a measurement instrument that is commonly used in monitoring and intervention research to assess how much people enjoy being physically active, as this has been related to physical activity adherence. However, while the measurement properties of PACES are well-researched in the English language, there is a gap of research in the German language, especially when looking at adults. Thus, the purpose of this work was to examine reliability, factorial validity, criterion-related validity, and measurement invariance across sex, age groups and time of the PACES for German-speaking adults. Data was obtained from the Motorik-Modul-Study (MoMo) in which 863 adults (53.5% female; mean age = 20.9 years) were examined. To investigate measurement invariance across age groups, data from 2,274 adolescents (50.5% female; mean age = 14.4 years) was obtained additionally. The study provided a nationwide representative sample for Germany. Results showed high internal consistency of PACES in adults (Cronbach’s α = .94). Confirmatory factor analyses confirmed the invariance of the measure across age groups, time, and sex. Criterion-related validity could be shown as the global factor significantly correlated with overall physical activity, physical activity in sports clubs, and leisure-time physical activity. The analyses of factorial structure indicated a method effect for positively and negatively worded items. Correlated uniqueness, latent method factor and a hybrid model were applied to analyze the method effect and results indicated that the method effect of positively worded items was predictive of physical activity independently of the global factor. Overall, it can be concluded that PACES is reliable, valid and invariant measure of physical activity enjoyment to be used in German-speaking adults. Further studies are warranted to examine the factorial structure of the PACES and the consequences of the method effect.

## Introduction

Physical activity (PA) is essential for adult’s health and well-being [1, 2], since it for example reduces risks to develop coronary heart disease, type 2 diabetes, breast and colon cancer, thus increasing life expectancy [3, 4]. With regard to psychosocial and mental health, PA has for example been evidenced to decrease anxiety and depression [5, 6], and to reduce the risk for Alzheimer disease [7]. For health-enhancing effects, it is recommended that adults engage in at least 150 minutes moderate-to-vigorous (MVPA) a week [8]. However, looking at PA levels in several countries [9, 10], the question how to improve PA adherence is a critical one [11, 12]. In this context, PA enjoyment has been shown to be an important factor theoretically and empirically [13].

Theoretically, the relationship between PA enjoyment and PA behavior can be derived from several theories [14, 15]. Over 120 years ago, Thorndike [16] stated the law of effect, which postulates that behavior producing a satisfying effect in a particular situation becomes more likely to occur again, and behavior that produces a discomforting effect become less likely to occur again. In accordance with the affect heuristic [17], feeling-as-information theory [18], and risk as feelings hypothesis [19], individuals make behavioral decisions by consulting their feelings. In exercise psychology, the achievement goal theory stresses the essential role of enjoyment for exercise adherence by considering the motivational climate [20, 21]. More recently, Brand and Ekkekakis [22] postulated in the affective-reflective theory the mechanisms how affective states might influence PA behavior.

Empirically, the association between PA enjoyment and PA behavior has been evidenced in several studies [23]. In a quasi-experimental study with adult participants, the control group received a standard exercise program, whereas in the intervention group, the instructors were trained to concentrate on promoting enjoyment. The intervention group showed increased re-attendance of the exercise classes compared to the control group [13].

In another study, participants aged between 50 and 79 years were categorized into inactive, active, and sustained maintainers regarding PA. It was investigated how six motives (weight / stress management, health & fitness, appearance, social / emotional benefit, enjoyment) differ across the three PA categories. Strongest differences were found for enjoyment, with the maintainers having significantly higher scores than the inactive ones [24]. This is supported by another study, showing that enjoyment seems to mediate exercise levels in adults [25]. In addition, in a qualitative study with 24 adult long-term PA maintainers (exercising longer than five years on a regular basis), all participants reported to enjoy PA [26].

A randomized-controlled trial (RCT) with 448 low-active adults who participated in a motivational PA intervention showed that PA enjoyment was an important predictor of six- and twelve-month PA and even more important than self-efficacy [27]. Williams and colleagues also conducted a RCT with 238 low-active adults, showing that the intervention effect was dependent on baseline PA enjoyment [28]. This is in line with a study by Kruk and colleagues [29], examining almost 900 adults and showing that PA enjoyment precedes MVPA and not vice versa, while another showed that PA enjoyment through social interactions is a motivational factor to be active for older adults [30].

These associations between PA enjoyment and PA engagement highlight the role of PA enjoyment for both promoting PA in adults, as well as for examining the relationship between PA enjoyment and other psychosocial constructs. For this purpose, it is important to have measurement instruments with tested validity and reliability [31]. In addition, measurement invariance is increasingly discussed as another measurement property to account for differences in interpretation and responses of the measurement instrument in different groups [32]. This is important to ensure that group differences are due to the construct in question and not due to differences in the understanding of the measurement instrument [33]. Vandenberg and Lance suggested five steps to test measurement invariance, including equivalence of structure, factor loading, measurement intercepts, structural covariance, and item errors (uniqueness) [32], which should be applied to measurement instruments of PA enjoyment.

A common measurement instrument to measure PA enjoyment and children and adolescents is the Physical Activity Enjoyment Scale (PACES), originally designed by Kendzierski and DeCarlo [34] with 18 items representing a scale between to bipolar adjectives (e.g. pleasant–unpleasant, enjoy–hate) on a 7-point scale. Motl and colleagues [35] revised the questionnaire by removing two items because of the lacking correspondence between items and the construct and redundancy of the items. The remaining 16 items were rephrased to improve comprehension of the questionnaire [35]. Measurement properties have been tested several times among children and adolescents for the English language [35–37], but only once for the German language [38].

Furthermore, measurement properties for PACES in adults are less well explored than in the younger age group, with heterogeneous results. The first PACES questionnaire was tested for reliability and validity with undergraduate students between 18 and 24 years, confirming the unidimensional structure [34]. Using Rasch models, the unidimensional structure of PACES was confirmed in a sample with adults aged 25 to 75 years, while item content did not fit all respondents [39]. In contrast, construct validity with 18 items could not be confirmed in a sample of older adults, but instead, an adapted scales with 8 items showed a good model fit together with measurement invariance across exercise groups and longitudinally [40]. However, some studies suggest that construct validity of PACES might be impacted by the valence of the items.

Two studies found that positively and negatively worded items shared unique variance, which could not be accounted for by a global factor [35, 38]. This issue questions the dimensional structure of PACES. Intuitively, the basic assumption was that both positively and negatively worded items are interchangeable and assess the same construct [34]. However, the results of confirmatory factor analyses consistently suggested a poor fit of a singe-factor model [35, 36]. Interestingly, a two-factor model led to an improvement of the model fit, whereas the overall fit was still not satisfying [36, 38]. Motl and Dishman [35] assumed that this misspecification was due to a method effect of positively and negatively worded items and proposed the strategy of correlated uniqueness (CU). This strategy to allow uniqueness terms to correlate amongst each other provided an almost perfect model fit in several studies [35, 38, 41]. However, the CU strategy has been criticized for comingling of unsystematic influences and method effects [42]. As an alternative, Bagozzi [43] proposed the latent method factor (LFM) strategy, which captures variance between items with the same method (i.e. positively and negatively worded items) by providing latent factors to each method effect. This strategy allows for direct estimation of both the global construct (i.e. enjoyment) and the method effects (of positively and negatively worded items) directly. Furthermore, it is possible to separate the error variance from the method variance, which was shown for Rosenberg's Self-Esteem Scale to be related to depression and life satisfaction [44]. However, LMF led in several cases to improper solutions, like negative variances and correlations estimates greater than 1.0 [41, 45]. In terms of PA enjoyment, the question arises whether the method effects provide additional predictive power for PA or other outcomes. In PA research, this issue has not been yet examined. In some studies, it has been supposed that the method effect is related either to positively or to negatively worded items [35], leaving the question open whether a hybrid model of CU and LMF might be a solution. Furthermore, while some studies in other areas suggest that method effects differ across groups, such as sex and age groups [45, 46], this has not been considered in the PACES studies up to date [35, 36, 38, 40].

In addition to the heterogenous study results regarding the measurement properties in adults of PACES, the measurement properties have never been tested in the German version of PACES. This version has only been validated for youth so far [38], based on an adapted 16-item scale of Motl and Dishman [35]. This is critical, given that research on PA enjoyment is prominent within the USA but lacking in European adults. Thus, this study aims to investigate a) reliability, b) construct validity, c) measurement invariance as well as d) criterion-related validity of PACES for German adults.

## Methods

### Participants

Participants were part of the national, representative Motorik-Modul-Study (MoMo) on PA and physical fitness in children and adolescents from Germany, and its umbrella study, the German Health Interview and Examination Survey for Children and Adolescents (KiGGS) [47, 48]. Study participants were selected based on a multi-stage sampling approach with two evaluation levels [49]. First, a systematic sample of 167 primary sampling units was selected from an inventory of German communities stratified according to the classification system that measures the level of urbanization and geographic distribution. Second, an age-stratified sample of randomly selected children and adolescents was drawn based on the official registers of local residents. Data for this study was obtained from Wave 1 (W1: 2009–2012) and Wave 2 (W2: 2014–2017). For Wave 1, 12,368 participants were part of the KiGGS measurement and 5,106 participants of the MoMo measurement. At Wave 2, 6,233 participants were randomly assigned from KiGGS to MoMo. As MoMo is a longitudinal cohort study, adolescents baseline participants transitioned into adulthood in Waves 1 and 2, resulting in the adulthood sample from which data was obtained for this study [50].

To be eligible for our study to test reliability, construct- and criterion-validity as well as measurement invariance for age and sex, participants must have been part of Wave 1 and must have been at least 18 years old. Data was obtained of participants that were at least 18 years at Wave 1. To examine if results of PACES in adolescence are comparable to the ones in adulthood via measurement invariance testing, an adolescent sample of the MoMo study was included cross-sectionally and longitudinally. To be eligible for our study to test measurement invariance across age groups, participants must have been part of Wave 1 and either in the adolescent age range (11–17 years) or adults (≥ 18 years). To be eligible for our study to test measurement invariance in the transition from adolescence to adulthood, participants were included that took part in both measurement occasions Wave 1 and Wave 2. In addition, participants had to be 11–17 years at the first measurement occasion (W1) and 18 years or older at the second measurement occasion (W2).

### Study design and procedures

For this study, a nationwide representative sample of children and adolescents from Germany was planned. Thus, a stratified, multi-stage sample with three evaluation levels was drawn [49]. First, a systematic sample of 167 primary sampling units was selected from an inventory of German communities stratified according to the BIK classification system that measures the level of urbanization and geographic distribution. Second, an age-stratified sample of randomly selected children and adolescents was drawn from the official registers of local residents for the KiGGS sample [49]. On the third level, MoMo participants were selected from the KiGGS sample [51].

The study was conducted according to the Declaration of Helsinki. Ethical approval was obtained by the Charité Universitätsmedizin Berlin ethics committee and the Federal Office for the Protection of Data. For both the MoMo- and the KIGGS-study, participants gave their written consent to participate and were informed in detail about the study and data management by the Robert Koch Institute. Parents gave their written consent for minors and the presence of a legal guardian was mandatory for participants aged below 15 years.

### Measures

#### Enjoyment

Enjoyment was measured using PACES. The adapted version has been developed by Motl and colleagues [35], consisting of 16 items (9 positive and 7 negative poled items) with responses on a 5-point Likert-Scale (1 = “*I disagree a lot*”; 5 = “*I agree a lot*”). All items were related to feelings concerning physical activity enjoyment suggesting face validity of the questionnaire. The items of the PACES seem to possess content validity. Item examples are “*When I’m physically active*, *I enjoy it”* (positively worded) and “*When I’m physically active*, *it’s not fun at all*” (negatively worded). For the overall scale, negatively worded items are recoded to fit with the positively worded scale. Then, the average of the sum of the items is calculated [36, 52]. The version of Motl and Dishman [35] has been translated into the German language which has already been reported elsewhere [38]. Briefly PACES was translated using forward and backward translation by qualified staff members (native speakers). Any wording differences were resolved by the translators. Comprehensibility of the items was assessed by 7^th^ grade students. The German version of PACES was then tested with around 700 adolescents in two studies [38]. Results showed that factorial validity and measurement invariance were inconsistent, but sufficient test-retest reliability (ICC = .76), internal consistency (α = .89), and criterion validity (r = .42 with PA diary and r = .16 with accelerometry data) were obtained [38].

#### Physical activity

Participants completed the MoMo Physical Activity Questionnaire [MoMo-PAQ; 53]. It consists of 28 items and measures frequency, intensity, time, and type of PA in four domains: PA at school, PA at organized sports clubs, PA outside of organized sports clubs, and daily PA. For those participants that were not in school any more, PA at the work place was assessed instead. Based on these four domains [38], an index was calculated considering moderate to vigorous PA (MVPA). Adults who were not in school were asked about activity at work. The outcome measure was MVPA minutes per week. Reliability and validity of this questionnaire were shown to be comparable to other international PA questionnaires [38].

### Statistical analysis

To investigate if the two-factorial structure is also appropriate for the German version of PACES, we first conducted an exploratory factor analysis (EFA). Following that, confirmatory factor analyses (CFA) with full-information maximum likelihood estimation was performed in AMOS 25 [54]. Through this method, less biased estimates are obtained compared to classical missing data procedures, including list-/pairwise deletion or mean imputation [55]. Across all three datasets, missing data ranged between 0.96%-0.98% for the PACES items.

Preliminary analyses revealed that negatively worded Item 7 (PA makes me depressed) and Item 12 (PA frustrates me) had extremely low means and standard deviations causing high skewness and kurtosis. The multivariate normality value and its critical ratio were 130.1 and 79.3, respectively, indicating nonnormality in the sample [56]. Therefore, we used the bootstrap method [57] to find approximate standard errors.

#### Reliability

Internal consistency was estimated using Cronbach’s α in SPSS 26 and by composite reliability. To calculate Cronbach’s α for the overall scale, negatively worded items were recoded to fit with the positively worded items. Cronbach’s α coefficient underestimates the reliability of the composite score due to the assumption of uncorrelated uniqueness among indicators, especially for multidimensional scales [58]. Based on the formula of Raykov [59], the composite score was estimated. All coeffcients are presented overall, by gender, and by age group.

#### Factorial validity

**Fig 1:**
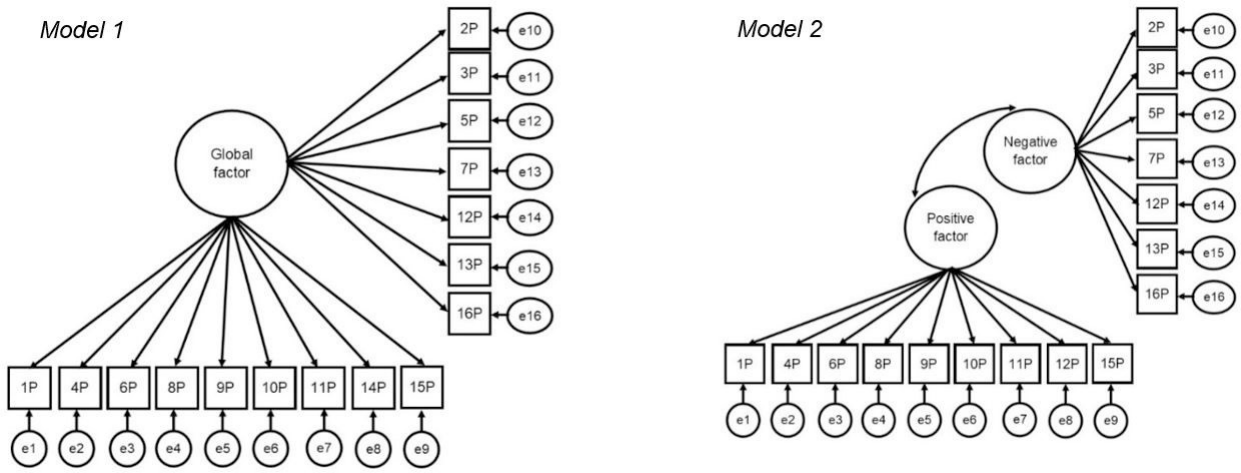
Structural equation models of PA enjoyment in adults. Model 1: one trait factor, no CU. Model 2: two trait factors, correlated positive and negative trait factors. P = positive item; N = negative items; e = error. Arrows at the errors represent correlations amongst all included items.

**Fig 2:**
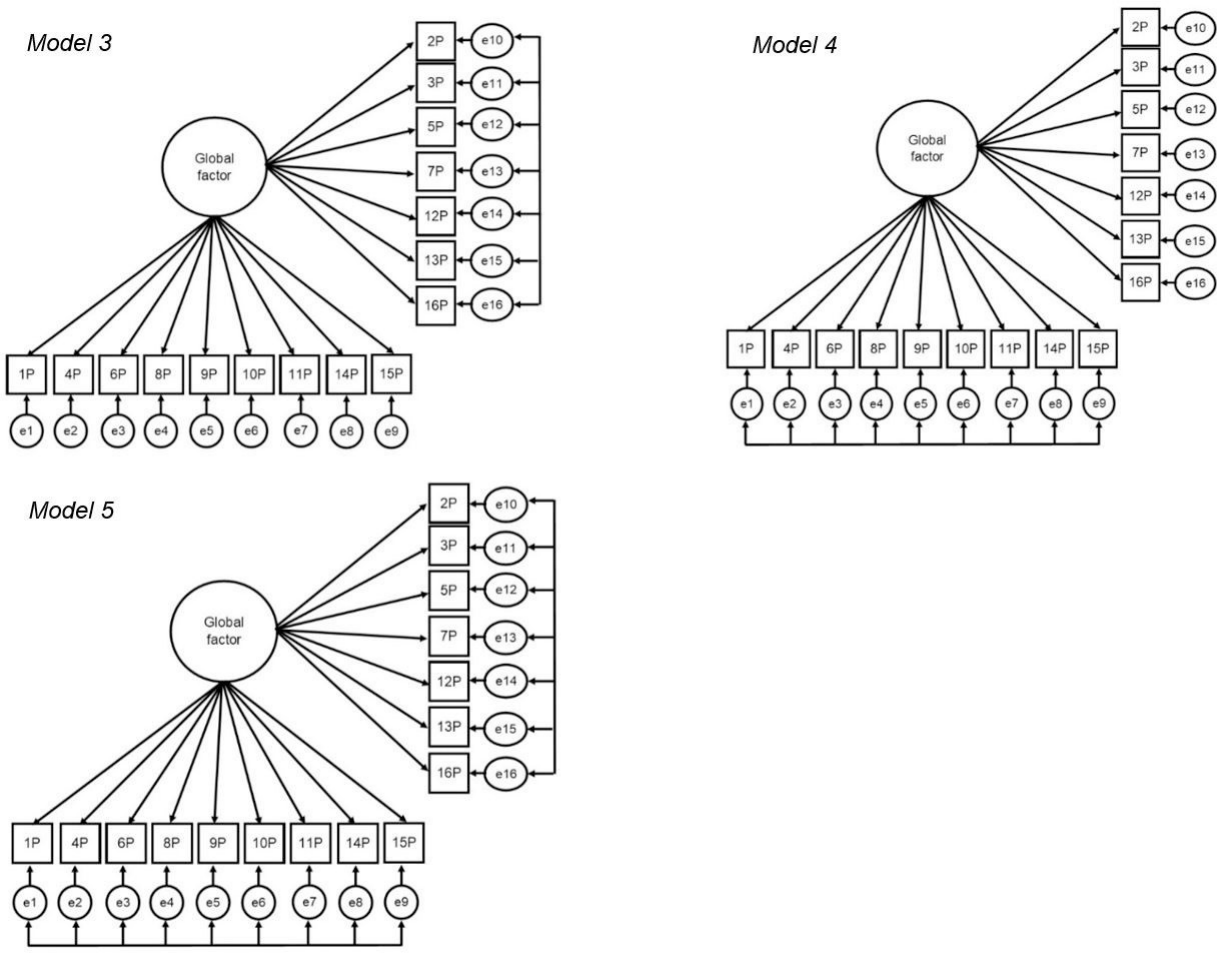
Structural equation models of PA enjoyment in adults for the CU framework. Model 3: one trait factor with CU among negative items. Model 4: One trait factor with CU among positive items. Model 5: One trait factor with CU among both positive and negative items. P = positive item; N = negative items; e = error. Arrows at the errors represent correlations amongst all included items.

**Fig 3:**
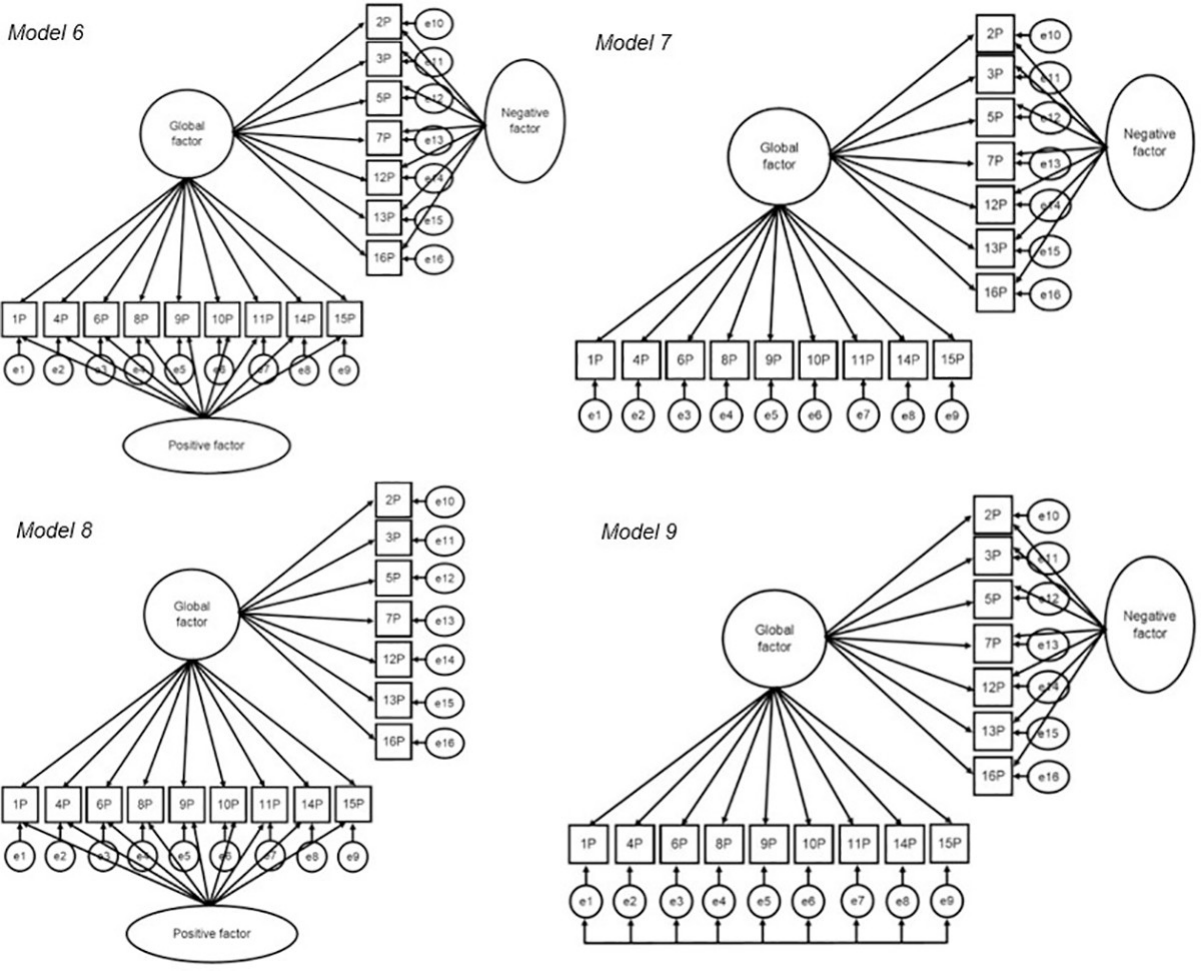
Structural equation models of PA enjoyment in adults for the LMF and combined CU / LMF hybrid framework. Model 6: one trait factor plus both a negative and positive LMF; Model 7; one trait factor and negative LMF; Model 8: one trait factor plus a positive latent method factor. P = positive item; N = negative items; e = error. Arrows at the errors represent correlations amongst all included items.

In order to examine the factorial validity of PACES, nine models were specified, following the approach of Marsh and colleagues [41] (Figs 1–3) and tested across sex and age groups. Model 1 suggests a global enjoyment factor without correlated error terms, thus not considering method effects, which is consistent with the version predominantly used in applied research. In Model 2, two latent factors are established, defined by negatively and positively worded items and without an overarching enjoyment factor (Fig 1). Models 3–5 (Fig 2) apply the CU framework by correlating the uniqueness terms. Model 3 posits one enjoyment factor with method effects (correlated uniqueness) among the negatively worded items. The same procedure is applied for Model 4 for the positively worded items. In Model 5, method effects are tested for negatively and positively worded items at the same time. For models 6–8 (Fig 3), the LMF strategy was applied. In Model 6, both positive and negative LMFs are included, in Model 7, only a negative LMF is included and in Model 8, only a positive LMF in included. Model 9 was a hybrid model of CU and LMF, in which the positive items are based on CU and negative items on LMF as preliminary analyses suggested that negatively worded items might predict PA independently of the global factor. A model of CU, a model of LFM and the hybrid model showing the best fit were chosen to test factorial validity, measurement invariance, and composite reliability.

Several fit indices were used to show the appropriateness of each model. The overall model fit was assessed using *χ*^*2*^-statistic, with a non-significant p-value indicating a good model fit [60]. However, the test depends on the study’s sample size [61]. Thus, even minor differences are detected between the model implied and observed covariance matrix so that model misspecifications are overestimated, leading to the rejection of the null-hypothesis [58]. The Comparative Fit Index (CFI) is used to show the relative fit improvement by comparing the suggested with the baseline model [62]. CFI values around .90 indicate an acceptable fit, values around .95 are considered as good fit [61, 62].

The Root-Mean-Square Error of Approximation (RMSEA) describes the error of approximation in the population, thus indicating the model’s closeness of fit. RMSEA values of ≤ .06 indicate a close and acceptable model fit. To show a good model fit, the zero should be contained in 90% confidence interval (CI) around the RMSEA point estimates [61, 63].

The successive, nested models were tested by *χ*^*2*^-difference tests. In addition to the model parsimoniousness and absolute fit, Akaikes information criterion (AIC) were used to determine the best fitting model as the models were not nested. Lower AIC values indicate a better model fit.

#### Measurement invariance

To investigate measurement invariance across age groups and gender, five nested models (Model A to Model E) were tested and compared using multiple group analysis [32, 64]. Each successive model consisted of the previous model restrictions and additional constraints [65]. The following components were consecutively tested: equivalence of structure (Model A), equivalence of factor loadings (Model B), equivalence of measurement intercepts (Model C), invariance of structural covariances (Model D), and invariance of item uniquenesses and correlations between uniquenesses (Model E) across time, sex, and age groups [32]. The models were tested for differences using *χ*^*2*^-statistic (Δ*χ*^*2*^). However, as the *χ*^*2*^-difference tests depend on sample size, differences of CFI (ΔCFI) were tested additionally [66]. The null hypothesis of invariance should be accepted if the *χ*^*2*^-difference test is not significant or ΔCFI ≤ 0.01 [66]. The significance level was set on 1%.

#### Criterion-related validity

To test the criterion-related validity of PACES, correlations between the PACES factors and MVPA overall, MVPA in sports clubs, and leisure MVPA were calculated in SPSS. For each latent factor, stability coefficients over a period of five years were used to estimate their systematic variance.

## Results

### Sample description

Participants or our cross-sectional adult sample in Wave 1 were between 18 and 26 years old (N = 863; 53.5% female; mean age = 20.88 years [SD = 1.87); BMI = 23.35 [SD = 4.41]). The adolescent sample that was obtained to test measurement invariance across age groups consisted of N = 2,274 participants (50.3% female; mean age = 14.38 [SD = 2.0]). The longitudinal sample that was obtained to investigate measurement invariance in the transition from adolescence to adulthood, consisted of N = 696 participants (55% female, mean age_W1_ = 15.16 years [SD = 1.59], mean age_W2_ = 20.56 years [SD = 1.66]).

### Reliability

Scale means, confidence intervals, standard deviations, Cronbach's α and composite reliabilities for Models 5, 6, and 9 are shown in Table 1. Cronbach's α of the questionnaire was 0.94, indicating very good internal consistency. The composite reliability of the global factor was 0.92–0.93, indicating very good reliability as well.

**Table 1:** Descriptive statistics and reliability of PACES.

	N	M (SD)	95% CI	α	α Pos. items	α Neg. items	Cr Model 5	Cr Model 6 (GF)	Cr Model 9 (GF)
Overall	863	4.19 (.61)	4.15–4.23	.94	.92	.88	.92	.92	.92
Females	462	4.16 (.61)	4.10–4.21	.94	.92	.88	.92	.92	.92
Males	401	4.24 (.62)	4.17–4.30	.94	.92	.88	.93	.93	.93

α = Cronbach’s alpha, cr = composite reliability; GF = global factor.

### Exploratory factor analysis (EFA)

An EFA with varimax rotation was performed on the cross-sectional adults sample to explore the factor structure of the 16 items. We applied three criteria to decide on the number of factors: eigenvalues >1 and factor loadings >.0.60. The above-listed criteria were met with the two-factor solution: 9 positivley worded items loaded on the first factors, 7 negatively worded items loaded on the second factor, which confirms that structure observed by previous studies [35, 38]. The first component had an eigenvalue of 8.31 and explained 51.96% of the variance, the second factor had an eigenvalue of 1.50 and explained 9.34% of the variance, resulting in 61.31% of the variance explained through the factors.

### Factorial validity

The results for factorial validity are presented in Table 2. The results for the single-factor model (Model 1) showed a poor fit (*χ*^*2*^_104_ = 1309.8; CFI = 0.856, RMSEA = 0.116; AIC = 1373.8). Model 2, which represents the two-factor model, indicated a better fit (*χ*^*2*^_103_ = 738.9; CFI = 0.923, RMSEA = 0.084; AIC = 804.9) than Model 1, but it was still not satisfactory. Model 3, which allowed for correlated uniqueness terms among negatively worded items, indicated a good model fit (*χ*^*2*^_83_ = 455.7; CFI = 0.955, RMSEA = 0.072; AIC = 561.7). In terms of AIC, it was superior to both Models 1 and 2. The single-factor model with CU terms among positively worded items (Model 4) also showed a good model fit (*χ*^*2*^_68_ = 411.3; CFI = 0.959, RMSEA = 0.076; AIC = 547.4). Model 5, which allowed for CU terms among positively and negatively worded items, provided an excellent model fit (*χ*^*2*^_48_ = 129.2; CFI = 0.990, RMSEA = 0.044; AIC = 305.2). This model was superior to all previous models. Model 6, which represents a model with both positive and negative LMFs, indicated a good model fit (*χ*^*2*^_88_ = 383.1; CFI = 0.964, RMSEA = 0.063; AIC = 479.1). Both Model 7 (negative LMF) and Model 8 (positive LMF) showed an inferior model fit (Model 7: *χ*^*2*^_97_ = 691.3; CFI = 0.928, RMSEA = 0.085; AIC = 769.3; Model 8: *χ*^*2*^_95_ = 732.5; CFI = 0.923, RMSEA = 0.089; AIC = 814.5). Among the LMF models, Model 6 showed the best fit with an AIC of 489.1. Model 9, the hybrid model of CU for positively worded items and LMF for negatively worded items provided an excellent model fit (*χ*^*2*^_61_ = 184.5; CFI = 0.985, RMSEA = 0.048; AIC = 332.5).

**Table 2:** CFA testing factorial validity of PACES.

	Model	*χ*^*2*^	df	p	CFI	RMSEA	AIC
*Overall*	Model 1	1309.8	104	< .01	.856	.116	1373.8
*Overall*	Model 2	738.9	103	< .01	.923	.084	804.9
*Overall*	Model 3	455.7	83	< .01	.955	.072	561.7
*Overall*	Model 4	411.3	68	< .01	.959	.076	547.4
*Overall*	Model 5	129.2	48	< .01	.990	.044	305.2
*Overall*	Model 6	383.1	88	< .01	.964	.063	479.1
*Overall*	Model 7	691.3	97	< .01	.928	.085	769.3
*Overall*	Model 8	732.5	95	< .01	.923	.089	814.5
*Overall*	Model 9	184.5	62	< .01	.985	.048	332.5
*Females*	Model 5	97.8	48	< .01	.989	.047	273.8
*Females*	Model 6	280.7	88	< .01	.956	.069	367.7
*Females*	Model 9	129.6	62	< .01	.985	.049	309.6
*Males*	Model 5	116.0	48	< .01	.983	.060	292.0
*Males*	Model 6	220.3	88	< .01	.966	.062	316.3
*Males*	Model 9	131.2	61	< .01	.984	.049	279.2

Note: *χ*^*2*^ = chi-square statistic; df = degrees of freedom, CFI = Comparative Fit Index; RMSEA = Root Mean Square of Approximation, AIC = Akaike Information Criterion.

The overall analysis showed that Models 5, 6 and 9 provide the best models fits with AIC = 336.3 for Model 5, AIC = 508.2 for Model 6 and AIC = 362.6 for Model 9. Thus, further analyses were conducted with the Models 5, 6 and 9. Amongst those three models, the model fits of Models 5 and 9 were superior to the fit of Model 6. All three models provided good fits for females and males. For the global factor, all factor loadings in the three models were significantly different from zero (Table 3). In Model 6, all factor loadings for the positive LMF were also significantly different from zero and had unexpectedly a negative sign. For the negative LMF in Models 6 and 9, the factors loadings of Item 7 (“It makes me depressed”) and Item 12 (“It frustrates me”) were dominant. Although the negative worded items were recoded into a positive direction, the factor loadings had surprisingly a negative sign in the Model 6, but a positive sign in the Model 9.

**Table 3:** Lambda loadings, standard errors (SE), and critical ratios (CR) for Models 5, 6, and 9 for the global factor.

		Model 5					Model 6						Model 9			
Item	λ		SE	CR		λ		SE	CR		λ			SE	CR	
**Global factor**																
1P	1					1					1					
4P	.97**		.04	23.64		.98**		.05	21.91		.97**			.04	23.50	
6P	.78**		.05	16.95		.76**		.04	17.17		.78**			.05	16.90	
8P	.92**		.04	21.51		.91**		.04	21.28		.92**			.04	21.34	
9P	.94**		.05	20.43		.90**		.04	20.69		.93**			.05	20.30	
10P	.94**		.05	19.37		.87**		.05	19.53		.93**			.05	19.90	
11P	.78**		.06	13.86		.72**		.05	13.91		.76**			.06	13.37	
14P	.85**		.05	15.75		.79**		.05	15.60		.83**			.05	15.39	
15P	.93**		.04	22.85		.89**		.04	23.06		.92**			.04	22.59	
2N	1					1.08**		.05	21.53		1.11**			.05	21.69	
3N	.93**		.04	22.08		.95**		.05	21.04		.98**			.05	21.16	
5N	.76**		.04	18.90		.53**		.04	12.96		.57**			.04	13.82	
7N	.42**		.03	12.43		.41**		.04	11.53		.44**			.04	11.88	
12N	.51**		.04	13.09		.84**		.04	20.05		.86**			.04	20.06	
13N	.90**		.04	20.56		1.00**		.05	22.38		1.02**			.05	22.09	
16N	1.05**		.05	21.49		1.05**		.05	23.07		1.07**			.05	22.78	
**Negative factor**																
2N						-.01		.03	-.48		-.01			.03	-.32	
3N						-.05		.03	-1.91		.03			.03	1.06	
5N						-.12**		.03	-4.41		.10**			.03	3.54	
7N						-.43**		.04	-9.53		.48**			.07	7.08	
12N						-.37**		.04	-8.49		.32**			.05	6.26	
13N						-.11**		.03	-3.68		.08**			.03	2.74	
16N						-.09**		.03	-2.94		.05			.03	1.73	
**Positive factor**																
1P						-.21**		.03	-8.04							
4P						-.27**		.03	-9.87							
6P						-.43**		.03	-14.98							
8P						-.32**		.03	-11.71							
9P						-.46**		.03	-16.83							
10P						-.51**		.03	-17.84							
11P						-.45**		.04	-12.74							
14P						-.41**		.03	-15.50							
15P						-.21**		.02	-17.68							

Note: **λ** = factor loadings; SE = standard error of lambda; CR = critical ratio; P = positively worded; N = negatively worded.

** p < .01

* p < .05.

### Measurement invariance

Measurement invariance was tested across sex, age groups, and across time for Models 5, 6, and 9. Results for Model 5 are reported in Table 4. The *χ*^*2*^-difference test was significant for the difference between Model B and Model C and between Model D and E for sex, age groups, and across time. However, the CFI did not decrease more than 0.01 for any of these comparisons. These results suggest that Model 5 can be regarded as invariant for sex, age groups, and across time.

**Table 4:** Analysis of invariance across sex, age, and time for Model 5.

Model	*χ*^*2*^	df	*p*	CFI	RMSEA	ΔCFI	*Δχ*^*2*^	Δdf	*p*
*Invariance by sex*									
Model A	212.5	96	< .01	.986	.038				
Model B	239.2	110	< .01	.984	.037	.002	26.7	14	> .01
Model C	282.6	126	< .01	.981	.038	.003	43.4	16	< .01
Model D	282.8	127	< .01	.981	.038	.000	.2	1	> .01
Model E	387.6	200	< .01	.977	.033	.004	104.8	73	< .01
*Invariance by age group (adolescents and adults)*									
Model A	308.8	96	< .01	.992	.027				
Model B	330.4	110	< .01	.992	.025	.000	21.60	14	> .01
Model C	441.0	126	< .01	.988	.028	.004	110.6	16	< .01
Model D	441.0	127	< .01	.988	.028	.004	0.0	1	> .01
Model E	680.7	200	< .01	.992	.028	-.004	239.7	73	< .01
*Invariance across time (adolescents and adults)*									
Model A	649.5	351	< .01	.975	.035				
Model B	686.5	365	< .01	.973	.036	.002	37.0	14	< .01
Model C	794.4	381	< .01	.965	.040	.008	107.9	16	< .01
Model D	859.0	439	< .01	.965	.038	.000	64.6	58	> .01
Model E	946.1	455	< .01	.959	.040	.006	87.1	17	< .01

Similar results were obtained for Model 6 and are presented in Table 5. The *χ*^*2*^-difference tests were significant for the differences between Model B and Model C and between Model D and E for sex, age groups, and across time. Additionally, the difference between Model C and Model D was significant for comparison between sex groups. However, again, the CFI did not decrease more than 0.01 for any of these comparisons. Therefore, these results suggest that Model 6 can be regarded as invariant for sex, age groups, and across time.

**Table 5:** Analysis of invariance across sex, age, and time for Model 6.

Model	*χ*^*2*^	df	*p*	CFI	RMSEA	ΔCFI	*Δχ*^*2*^	Δdf	*p*
	*Invariance by sex*								
Model A	498.3	176	< .01	.961	.046				
Model B	551.0	207	< .01	.959	.044	.002	52.7	31	< .01
Model C	599.5	223	< .01	.955	.044	.004	48.5	16	< .01
Model D	599.6	224	< .01	.955	.044	.000	0.1	1	> .01
Model E	639.2	240	< .01	.952	.044	.003	39.6	16	< .01
	*Invariance by age group (adolescents and adults)*								
Model A	980.5	176	< .01	.970	.038				
Model B	1022.7	207	< .01	.970	.035	.000	42.2	31	> .01
Model C	1143.0	223	< .01	.966	.036	.004	120.3	16	< .01
Model D	1143.1	224	< .01	.966	.036	.000	.01	1	> .01
Model E	1281.1	270	< .01	.961	.037	.005	138.0	16	< .01
	*Invariance across time (adolescents and adults)*								
Model A	976.0	431	< .01	.954	.043				
Model B	1004.5	459	< .01	.954	.042	.000	28.6	28	> .01
Model C	1114.7	475	< .01	.946	.045	.008	110.2	16	< .01
Model D	1116.6	476	< .01	.946	.045	.000	1.9	3	> .01
Model E	1236.2	492	< .01	.937	.047	.009	119.6	16	< .01

The results for Model 9 are presented in Table 6. The *χ*^*2*^-difference tests indicated that the differences between Model B and C as well as between Model D und E for all three comparisons across age groups, sex, and time were significant. However, once more, ΔCFI did not exceed .01 for any of these comparisons. Therefore, Model 9 can also be regarded as invariant across sex, age groups, and time.

**Table 6:** Analysis of invariance across sex, age, and time for Model 9.

Model	*χ*^*2*^	df	*p*	CFI	RMSEA	ΔCFI	*Δχ*^*2*^	Δdf	*p*
*Invariance by sex*									
Model A	281.6	124	< .01	.981	.038				
Model B	314.6	146	< .01	.980	.037	.001	33.0	22	> .01
Model C	358.6	162	< .01	.976	.038	.004	44.0	16	< .01
Model D	358.9	163	< .01	.976	.037	.000	.03	1	> .01
Model E	441.8	214	< .01	.972	.035	.004	82.9	51	< .01
*Invariance by age group (adolescents and adults)*									
Model A	525.8	124	< .01	.985	.032				
Model B	565.4	146	< .01	.984	.030	.001	39.6	22	> .01
Model C	679.2	162	< .01	.981	.032	.003	113.8	16	< .01
Model D	679.3	163	< .01	.981	.032	.000	.01	1	> .01
Model E	880.2	214	< .01	.975	.032	.006	200.9	51	< .01
*Invariance across time (adolescents and adults)*									
Model A	1462.9	378	< .01	.908	.037				
Model B	1485.5	399	< .01	.908	.036	.000	22.6	21	> .01
Model C	1582.8	415	< .01	.901	.039	.007	97.3	16	< .01
Model D	1634.3	452	< .01	.900	.038	.001	51.5	37	> .01
Model E	1710.0	468	< .01	.896	.040	.004	75.7	16	< .01

### Criterion-related validity

The results of criterion-related validity are presented in Table 7. For Model 5, the global factor was significantly (all p < .01) correlated with overall PA (r = .43), PA in sports clubs (r = .34) as well as PA in leisure time (r = .26). For Model 6, the global factor significantly correlated with overall PA (r = .33), PA in sports clubs (r = .27) and PA in leisure time (r = .20) (all p < .01). The latent factor for positively worded items significantly, but negatively correlated with all three indicators of PA. Finally, the latent factor for negatively worded items significantly positively correlated with overall PA and PA in sports clubs, but not with leisure time PA.

**Table 7:** Standardized regression weights between PA domains and global / latent factors.

PA	GF	PosF	NegF	R^2^
*Model 5*				
Overall	.43**			.19
Sports club	.34**			.11
Leisure	.26**			.07
Stability	.62**			
*Model 6*				
Overall	.33**	-.30**	.17**	.23
Sports club	.27**	-.22**	.16**	.15
Leisure	.20**	-.21**	.03	.09
Stability	.57**	.31**	.32**	
*Model 9*				
Overall	.39**		-.20**	.19
Sports club	.31**		-.18**	.12
Leisure	.23**		-.05	.06
Stability	.56**		.80**	

Note

** = p < .01, GF = global factor, PosF = positively worded enjoyment; NegF = negatively worded enjoyment.

For Model 9, the global factor significantly correlated with overall PA (r = 0.39), PA in sports clubs (r = 0.31) and PA in leisure time (r = 0.23) (all p < .01). The latent factor for negatively worded items showed correlations with overall PA (r = -0.20) and PA in sports clubs (r = -0.18), but not with PA in leisure time. The amount of explained variance was highest for Model 6 explaining 23% in overall PA, 15% in PA in sports clubs and 9% in leisure time PA.

The stabilities for a period of five years were significant for all latent factors in all models. The stabilities of the global factor ranged between .56 and .62. The stability of the LMF for positively worded items was .31 in Model 6 and the stability of the LMF for negatively worded items was .32 in Model 6 and .80 in Model 9, suggesting that LMFs also contain parts of the variance, which are stable over time.

## Discussion

This study aimed to investigate reliability, construct validity, measurement invariance, and criterion validity of the German version of the PACES.

### Psychometric properties of PACES

The results of this study suggest that the German version of PACES is a very reliable measure of PA enjoyment in adults. The coefficients of Cronbach´s alpha and composite reliabilities are well above 0.90. Comparable results were found in a sample of US adults [35] as well as German adolescents [38].

Concerning the invariance of PACES, significant deviations were found for equivalence of measurement intercepts (Model C) and invariance of item uniquenesses and correlations between uniquenesses (Model E) according to the *χ*^*2*^-difference tests. These deviations were found for all tested models across age groups, sex, and time. However, due to the hypersensitivity of the *χ*^*2*^-difference test, the criterion of ΔCFI ≤ 0.01 [66] was applied. In this sense, there were no substantial deviations of the supposed invariance assumptions. Therefore, PACES is invariant across age groups, sex, and time.

### Criterion-related validity

The results of the criterion-related validity support the assumption that PACES is a predictor of PA in adults. The global factor was related to overall PA, PA in sports clubs and leisure time PA. Additionally, the positive LMF significantly correlated with all three indicators of PA and the negative LMF significantly correlated with overall PA and PA in sports clubs. These results mean that both positive and negative LMFs are independent predictors of PA. Comparing the amount of explained variance of Model 6 with that of Model 5 and 9, we conclude that the positive LMF explains additional 4% of the variance in overall PA, 4% in PA in sports clubs and 2% in leisure time PA. However, the comparison between Model 9 and Model 5 does not suggest that the negative LMF substantially contributes to the explanation of the variance in PA. Thus, only the positive LMF has incremental predictive power for PA.

Interestingly, the correlation between positive LMF and PA in Model 6 was negative, suggesting that the positive LMF explains some negative aspects of PA. This might be due to suppression effects that cause the shift of the sign of the factor loadings in the LMFs. The global factor represents general enjoyment in PA, whereas the positive and negative LMFs represent the remaining systematic variance. The sign of the loadings can change according to the configuration of the model and its relationship with the dependent variable. Furthermore, it is possible that the LMFs represent some negative aspects of PA enjoyment such as tediousness and frustration, which might not only be the opposite of enjoyment but rather suggest that enjoyment of PA and some negative emotions exist simultaneously. One possible way to explore the real meaning of the LMFs would be to employ, simultaneously to PACES, further measurement instruments measuring negative affective states during PA and to examine its interplay with PA.

In general, the results of this study suggest that both LMFs contain systematic parts of the variance, which can predict PA independently of the global factor. This idea is supported by the fact that both LMFs have significant stability coefficients over a period of five years. Therefore, the method effects of positively and negatively worded items on PA are systematic and need to be considered when predicting PA.

### Factorial structure

Regarding the factorial structure of the PACES, the results suggest that a method effect of negatively and positively worded items exists. The CU and the LMF strategy have been proposed to deal with method effects [43]. Several variations of these strategies were shown to have better model fits than the pure single-factor model and the two-factor model. Among the CU models, Model 5, which allowed for correlated uniqueness terms among positively and negatively worded items, showed the best fit. Among the LMF models, Model 6, which postulates both a positive and a negative LMF, was shown to be superior over models with only one LMF. These results suggest that the method effect separately influences both positively and negatively worded items. Furthermore, a hybrid model with CU for positively worded items and LMF for negatively worded items provided a substantially better model fit than Model 6 with two LMFs, but a slightly worse model fit than Model 5 with CU for both positively and negatively worded items.

Regarding the model’s fit, one should prefer Model 5 with CU for positively and negatively worded items. Regarding the criterion-related validity, however, one should take into consideration that both positive and negative LMFs were significantly related to the indicators of PA and had a moderate stability over a period of five years. According to Lance and colleagues [42], the LMF strategy should be preferred due to the advantages derived from the parameterization of method effects in the LMF. In this study, the hybrid model did not show to be an alternative to CU and LMF. CU provided a considerably better model fit and the model with two LMFs explained significantly more variance in PA than the hybrid model. According to the results of our study, the model with a positive and a negative LMF should be preferred compared to the CU and the hybrid model.

### Implications for practice and science

The results of this study suggest that the German version of PACES is a reliable, valid and invariant measure of PA enjoyment in adults. It can be used in large population studies to examine and compare the levels of PA enjoyment across different population groups. The questionnaire is suitable for testing differences between specific population groups (e.g. age groups, sex). Furthermore, the PACES can be applied to examine intrapersonal changes in PA enjoyment due to its time invariance. Therefore, the PACES can be used to examine how interventions impact PA enjoyment. However, further research is required to deepen our knowledge on factorial structure of the questionnaire and to examine the predictive properties of the scale for PA behavior. Especially, the role of the positive and negative LMFs needs to be clarified in further studies.

### Strengths and limitations

This study has several strengths. First, the sample of the MoMo-Study is a large and representative of German adolescents and young adults. Second, it provides longitudinal data, allowing to examine the invariance across time. Third, sophisticated statistical analyses using structural equation modelling were conducted to examine the psychometric properties of the measurement instrument. However, this study has also some limitations. This study does not contain data to estimate test-retest reliabilities of the PACES. Furthermore, a study including other related affective constructs (e.g. tediousness, stress or frustration) should be conducted to examine the relative meaning of the LMFs.

## Conclusion

The German version of the PACES is a reliable and valid measurement instrument of PA enjoyment in adults. Furthermore, the measure is invariant across age groups, sex, and time. Therefore, the PACES can be used in population studies to compare the PA enjoyment of different population groups to examine the need for interventions as well as in intervention studies to examine the effectivity of PA interventions. The results of this study suggest that the PACES is associated with method effects for positively and negatively worded items, which need to be further examined in future studies.
